# Pilot Transcriptomic Profiling of Canine Oral Melanoma Reveals Conserved Oncogenic Pathways and Uncharacterized Molecular Signatures

**DOI:** 10.3390/cancers17132106

**Published:** 2025-06-23

**Authors:** Carmen G. Pérez-Santana, Francisco Rodríguez-Esparragón, Sara E. Cazorla-Rivero, Ana A. Jiménez-Alonso, Bernardino Clavo, Jesús M. González-Martín, Ángeles Cánovas-Molina, Carmen Bartolomé, Lidia Estupiñán, Enrique Rodríguez Grau-Bassas

**Affiliations:** 1 Unidad de Investigación, Hospital Universitario de Gran Canaria Doctor Negrín, 35019 Las Palmas de Gran Canaria, Spain; scazorla@ull.edu.es (S.E.C.-R.); ana.jimenez114@alu.ulpgc.es (A.A.J.-A.); bernardinoclavo@gmail.com (B.C.); josu.estadistica@gmail.com (J.M.G.-M.); canovaspi@hotmail.com (Á.C.-M.); cbardur@gobiernodecanarias.org (C.B.); lestqui@gobiernodecanarias.org (L.E.); 2 Fundación Canaria Instituto de Investigación Sanitaria de Canarias (FIISC), 35012 Las Palmas de Gran Canaria, Spain; enrique.rodriguez@ulpgc.es; 3 Instituto Universitario de Sanidad Animal y Seguridad Alimentaria (IUSA), Campus of Arucas, Las Palmas de Gran Canaria University, 35001 Las Palmas de Gran Canaria, Spain; 4 Instituto Universitario de Enfermedades Tropicales y Salud Pública de Canarias de la Universidad de La Laguna, 38296 La Laguna, Spain; 5 CIBER de Enfermedades Infecciosas, Instituto de Salud Carlos III, 28029 Madrid, Spain; 6 Radiation Oncology Department, Hospital Universitario Doctor Negrín, 35019 Las Palmas de Gran Canaria, Spain; 7 Spanish Group of Clinical Research in Radiation Oncology (GICOR), 28290 Madrid, Spain

**Keywords:** canine oral melanoma, RNA sequencing (RNA-seq), differentially expressed genes (DEGs), comparative oncology, melanoma biomarkers

## Abstract

Background: Canine oral melanoma (COM) is an aggressive and often fatal neoplasm in dogs, with clinical and molecular similarities to human melanoma. Despite its relevance as a comparative oncology model, the molecular mechanisms underlying COM remain poorly understood. This study aimed to characterize gene expression profiles in COM to identify differentially expressed genes (DEGs), potential biomarkers, and therapeutic targets. Methods: In this pilot study, we performed RNA sequencing (RNA-seq) on tumor and healthy oral tissue samples from dogs. Two independent analytical pipelines—Bowtie2-DESeq2 and HISAT-StringTie-Ballgown—were used to ensure robustness in DEG detection. We also conducted pathway enrichment and isoform-level analyses to investigate biological processes and alternative splicing events. Results: Both approaches identified a core set of 929 common DEGs. Key oncogenic pathways, including MAPK/ERK and cell cycle regulation, were significantly affected, with notable upregulation of BRAF, NRAS, CDK4, and MITF (log2FC = 2.86, *p* < 0.001). The transcription factor SOX10 and the cytokine IL-33, both previously implicated in melanoma progression, were consistently overexpressed. Additionally, NF1, a known RAS pathway inhibitor, was also upregulated. Isoform analysis revealed novel transcript variants, suggesting a complex layer of post-transcriptional regulation in COM. Many DEGs remained uncharacterized, and chromosomal distribution analysis highlighted potential genomic influences. Conclusions: Our findings provide new insights into the molecular landscape of COM, reinforcing its utility as a model for human melanoma. The identification of conserved oncogenic pathways and novel transcript variants opens avenues for further functional studies and the development of targeted therapies in both veterinary and human oncology.

## Simple Summary

Canine oral melanoma is an aggressive cancer with limited treatment options and poor prognosis. It shares key biological features with human melanoma, making it a valuable model for cancer research. In this study, we analyzed gene expression in tumor and healthy tissues from dogs to identify molecular alterations associated with this disease. We found that several genes related to immune response, cell proliferation, and tumor progression were highly expressed in tumors, while genes linked to normal epithelial structure were markedly reduced. A large proportion of differentially expressed genes remain uncharacterized, suggesting that critical biological pathways in this cancer are still unknown. We also observed an asymmetric chromosomal distribution of gene expression changes, possibly reflecting underlying genomic features. These findings offer new insights into the molecular landscape of canine oral melanoma and highlight potential biomarkers and therapeutic targets, reinforcing the utility of this model for advancing research in both veterinary oncology and comparative studies with human melanoma.

## 1. Introduction

Malignant melanoma (MM) is the most frequently documented oral malignant tumor in dogs, whereas in humans, it is a rare but aggressive cancer [1,2]. Canine oral melanoma (COM) constitutes a MM form with aggressive characteristics. It is a tumor with high metastatic potential and high local invasiveness [3]. The most common sites of metastasis include regional lymph nodes and the lungs. The percentage of involvement of these locations in metastasis ranges from 30.3% to 74.0% at the level of the regional lymph nodes, as well as from 14.0% to 92.0% for distant metastatic spread to the lungs and other organs [4]. The cause of death described in the majority of dogs with this tumor is distant metastasis rather than local recurrence. COM shares notable resemblances with Human Mucosal Melanoma (HMM), its human counterpart in clinical presentation, histopathological features, and overall biology. Therefore, despite the different susceptibility reported according breeds [5], the domestic dog has evolved to be an important biomedical model for studies regarding the genetic basis of disease, morphology, and behavior [1]. 

Previously reported genetic studies in dogs have relied, for years, on a draft reference genome of a purebred female boxer dog named “Tasha” published in 2005. The original draft derived from a Sanger whole genome shotgun sequencing approach and although have been constantly updated, it contained gaps, assembly errors, and missing sequences. Jagannathan et al. [6] presented in 2021 the Dog10K_Boxer_Tasha_1.0, an improved chromosome-level highly contiguous genome assembly of Tasha that increased sequence contiguity > 100-fold, closed >23,000 gaps of the CanFam3.1 reference assembly and improved gene annotation by identifying >1200 new protein-coding transcripts.

We have previously reported varying outcomes in COM developing amelanotic melanomas and melanotic melanomas, with some dogs completing one year without recurrence, while others experienced progressive disease, leading to several COM-related deaths

In this pilot study we aimed to identify differentially expressed genes in COM using transcriptomic analysis, which may shed light on the molecular mechanisms underlying tumor progression and metastasis.

## 2. Materials and Methods

### 2.1. Sample Collection, Processing, and Bioinformatics Analysis

Tissue biopsy samples from dogs with oral melanoma were collected for this study at the Veterinary Oncology Service of GICOREC IUSA (Gran Canaria, Spain) of the Universidad de Las Palmas de Gran Canaria (ULPGC). The recruitment period spanned from 2021 to 2023, with animals followed up for one year. The dogs were presented for surgical treatment and were managed according to Good Clinical Practice guidelines for animal clinical studies. The study was approved by the Bioethics Committee of ULPGC (OEBA-ULPGC 33/2020R1). Owners were informed and gave their consent. Tissue processing for total RNA extraction, bioinformatics analyses, and validation experiments were carried out at the Research Unit of the Hospital Universitario de Gran Canaria Dr. Negrin (HUGCDN).

A total of 20 samples and 10 unrelated control oral skin samples were obtained following surgical resection. Transcriptomic analysis was ultimately performed on 6 COM samples and 6 control oral skin samples. Total RNA was extracted from tumor and healthy tissue samples using the modified TRIzol method with DNase I treatment. One microgram (μg) of total RNA was used to prepare libraries with the TruSeq Stranded Total RNA Library with Ribo-Zero Gold, followed by sequencing on a NovaSeq 6000 platform with paired-end 150 bp reads (2 × 150 bp) at a depth of 60 million reads per sample. Library preparation and transcriptome sequencing were performed at Macrogen. Quality control was assessed using high-sensitivity chips on an Agilent Bioanalyzer (Agilent, Santa Clara, CA, USA) before sequencing.

Quality control was performed using FastQC and preprocessing of sequencing reads were performed using FastQC and Rfastp. To ensure robust and comprehensive data analysis, we employed two complementary RNA-seq analysis pipelines as follows:

1. Read alignment and gene-level quantification: Reads were aligned using Bowtie2 and the Rsubread package, and gene counts were obtained using the summarizeOverlaps function from the GenomicAlignments package. Differential expression analysis was conducted using DESeq2. Transcript-level assembly and quantification: Reads were aligned using HISAT, assembled into transcripts with StringTie, and quantified using Ballgown. The results were then contrasted using DESeq2 to assess differential expression.
2. The rationale for using these two approaches was to ensure a comprehensive analysis of gene expression. The Bowtie2 pipeline provides a traditional read count-based approach, which is efficient for gene-level expression analysis. In contrast, the HISAT/StringTie/Ballgown pipeline allows for transcript reconstruction, facilitating the detection of novel isoforms and alternative splicing events. By integrating both a gene-level and a transcript-level approach, we aimed to enhance the robustness of our analysis, ensuring the detection of both differentially expressed genes and novel isoforms.

The *Canis lupus familiaris* genome and annotation files (GCF000002285.5, canFam6) assembly [7] were obtained from NCBI for reference-based analysis.

Principal Component Analysis (PCA) of melanoma and healthy control tissue samples was visualized using R (version 4.3.2) and Bioconductor software. Mean-Average (MA) plots, volcano plots for differentially expressed genes (DEGs), and heatmaps of highly expressed genes were also generated using these frameworks.

### 2.2. Gene Set Enrichment Analysis and Pathways Analysis (KEGG)

Gene Set Enrichment Analysis (GSEA) was performed using the ClusterProfiler software (version 4.6.2) [8] with the gene set identifier obtained from the org.Cf.eg.db package (version 3.16.0) and the DOSE (version 4.5) [9]. The enrichGO function was used to analyze the overrepresentation of significant genes in Gene Ontology terms, while the gseKEGG function was applied to assess gene list distribution in KEGG pathways. Data were visualized using dot plots and cnetplots, respectively.

### 2.3. RT-qPCR

cDNA was synthesized from 1 μg of total RNA using the iScript^TM^ cDNA synthesis kit according to the manufacturer’s protocol. The qPCR was performed using a SsoFast EvaGreen qPCR Master Mix Universal kit and a CFX Real-Time PCR System. The primer sequences used in this study are available upon request.

## 3. Results

### 3.1. Main Patient Characteristics

Table 1 summarizes the main clinical and demographic characteristics of the dogs included in this study. The cohort consisted of twelve animals as follows: six healthy controls and six dogs diagnosed with oral malignant melanoma. The control group included a mix of breeds, ages ranging from 5 to 11 years, and both sexes. The melanoma group comprised animals of various breeds, with tumors located primarily in the lip, mandible, cheek, and maxilla. Tumor sizes ranged from 1.5 cm to 7.7 cm, and clinical staging based on the TNM system (Tumor–Node–Metastasis) classified cases from Stage I to Stage III, indicating variable degrees of disease progression. Independent of the presence of regional lymph node metastases (most of which were reactive lymphoid hyperplasia), both the lymph nodes and the primary tumor were removed during the same surgical procedure. However, none of the patients showed evidence of distant lung metastases at the time of the study.

### 3.2. RNA-Seq Analysis

RNA-seq was successfully performed on all samples included in the study. Sequence data of 2 × 150 bp in length with a Phred score > 30 were selected for alignment. The total number of read pairs ranged from 33 to 174 million. The mean percentage of alignments to the canFam6 reference genome was 88%, with no significant difference between COM and control samples (*p* > 0.05).

### 3.3. Principal Component Analysis (PCA)

**Figure 1:**
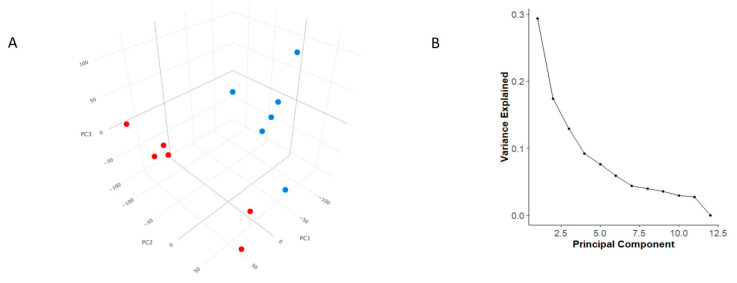
Principal Component Analysis and Scree Plot. (**A**) Principal Component Analysis (PCA) showing clear clustering of sample groups based on condition. (**B**) Scree plot showing the percentage of variance explained by each principal component. The first two components capture the majority of the variance, supporting the separation observed in the PCA plot.

Principal Component Analysis (PCA) based on gene expression profiles revealed that PC1 and PC2 together explained 45.7% of the total variance, which is close to the typical threshold of acceptability around 40–50% of the variance explained by the first two component. Including PC3 and PC4 increased the explained variance to 68.8% (Figure 1A). Since PCA mainly shows how samples are distributed, a scree plot was drawn to complement the PCA interpretation. As depicted in Figure 1B, there was a steep slope at the beginning, indicating that the first principal components (PC1 and PC2) explain most of the variability. However, the subsequent components provide increasingly less relevant information only after the inflection point, approximately located between PC3 and PC5.

### 3.4. Differentially Expressed Genes (DEGs)

Using the Bowtie2-DESeq2 workflow, we identified 35,555 differentially expressed genes (DEGs). Of these, 1.99% lacked an ENTREZID annotation, meaning that 35,485 DEGs had corresponding SYMBOL and ENTREZID annotations. After removing duplicates and addressing missing values, we retained 32,283 genes for enrichment analysis, of which 31,766 had defined ENTREZID annotations, while 517 genes remained unannotated.

**Figure 2:**
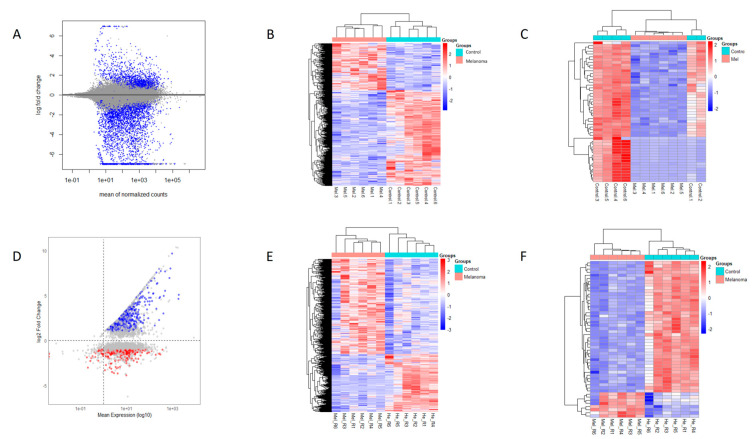
Differential expression analysis results using two RNA-seq workflows. (**A**) MA plot showing the relationship between the mean of normalized counts and the log2 fold change, obtained using the Bowtie2–DESeq2 pipeline. (**B**) Heatmap of gene expression across melanoma and control samples. (**C**) Heatmap of the top 50 differentially expressed genes (DEGs). (**D**–**F**) Corresponding plots generated using the HISAT2–StringTie–Ballgown workflow, providing a comparative visualization of expression differences across workflows.

Among the 15,834 upregulated genes, 6835 were not fully characterized, whereas 8057 out of 15,932 downregulated genes also lacked full characterization. Figure 2A shows a Mean Average (MA) plot displaying the relationship between the mean of normalized counts (*x*-axis) and the log fold change (*y*-axis). The distribution of points suggests that most genes do not exhibit significant changes, while some differentially expressed genes are highlighted in blue. Figure 2B shows a heatmap of gene expression across melanoma and control samples. The hierarchical clustering effectively separates the two conditions, indicating distinct expression profiles.

**Figure 3:**
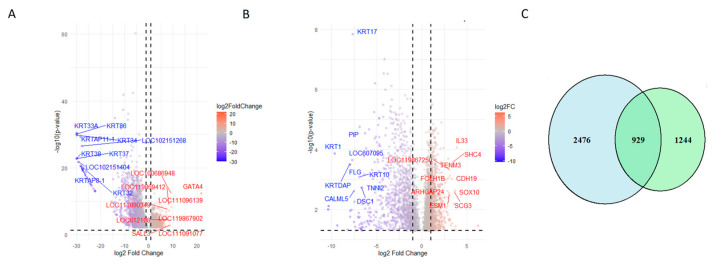
Comparison of differentially expressed genes identified by both workflows. (**A**) Volcano plot showing the distribution of DEGs identified using the Bowtie2–DESeq2 pipeline. The top 10 upregulated and downregulated genes are labeled. (**B**) Volcano plot for DEGs identified using the HISAT2–StringTie–Ballgown pipeline, highlighting the top 10 upregulated and downregulated genes. (**C**) Venn diagram depicting the overlap of DEGs detected by both workflows, indicating the shared and unique transcriptomic changes captured by each approach.

Applying a stringent threshold of |logFC| > 1 and *p* < 0.05, we identified 3219 DEGs as follows: 973 upregulated and 2246 downregulated. Table 2 presents the chromosome location and mean expression of the top identified DEGs in canine oral melanoma (COM) and healthy control tissue samples, while Table 3 lists top 10 upregulated and top 10 downregulated DEGs resulting from the DESeq2 analysis. A heatmap of the top 50 DEGs (Figure 2C) further confirms the separation of COM and control samples, with some melanoma samples showing variability that may suggest subtypes or biological heterogeneity. Figure 3A presents a volcano plot highlighting the top 10 upregulated and downregulated DEGs after DESeq2 analysis.

### 3.5. Comparison with HISAT-StringTie-Ballgown Pipeline

A similar analysis using the HISAT-StringTie-Ballgown pipeline identified a comparable number of DEGs. After applying filtering criteria, 12,883 DEGs remained. From these, 3357 genes were selected based on their *p*-values, and missing values were removed, resulting in 2173 retained DEGs. Using |logFC| > 1 and *p* < 0.05, a total of 276 upregulated and 383 downregulated DEGs were found. Notably, differences in the top deregulated genes were observed between this pipeline and the Bowtie2-DESeq2 approach.

Variance thresholding was applied to focus on genes with high expression variability, enhancing the identification of biologically relevant DEGs. Figure 2D–F illustrate the distribution of expression data and heatmaps of the most significant DEGs. Table 4 lists the top 10 upregulated and downregulated DEGs from the HISAT-StringTie-Ballgown analysis, while Figure 3B shows a volcano plot highlighting key DEGs.

To assess overlap between both pipelines, a Venn diagram (Figure 3C) revealed 929 common DEGs, representing 27% of the DESeq2 results and 43% of the Ballgown results. This overlap underscores a core set of genes consistently deregulated, despite methodological differences.

### 3.6. Chromosomal Distribution of DEGs

**Figure 4:**
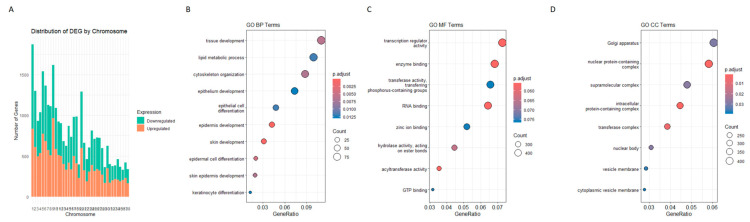
Functional and chromosomal distribution of DESeq2-derived DEGs. (**A**) Chromosomal distribution of DEGs identified through the DESeq2 pipeline. DEGs are mapped along human chromosomes revealing positional enrichment. (**B**–**D**) Gene Ontology (GO) enrichment analysis classifying DEGs into three functional categories as follows: Biological Process (BP), Cellular Component (CC), and Molecular Function (MF). Bar plots show the top enriched terms in each category.

Chromosomal distribution analysis of DESeq2-identified DEGs revealed an increasing trend in DEG count with increasing chromosome size (Figure 4A). Smaller chromosomes (29–38) contained fewer DEGs, whereas chromosome 1 had the highest count (1036 downregulated, 838 upregulated). Chromosome 9 exhibited the highest number of upregulated genes (968), suggesting its potential involvement in COM pathogenesis. Chromosomes 5 and 20 also showed strong differential regulation, with similar patterns observed in Ballgown results.

### 3.7. Human Oncogenes in the Dataset

A list of human cutaneous melanoma oncogenes was cross-referenced with the obtained DEGs. The MAPK/ERK pathway regulator BRAF was included, as mutations in the V600E variant are present in approximately 50–60% of human cutaneous melanomas [10]. The oncogenic GTPase NRAS, whose mutation in Q61K/R/L accounts for 20–30% of cases [11]. The KIT receptor tyrosine kinase and the transcription factor MITF, both amplified in 10–20% of melanomas were also considered [12]. In addition, CDK4 and CCND1, cell cycle regulators that are frequently mutated or amplified in BRAF wild-type melanomas, were included [10]. Regarding tumor suppressors, CDKN2A, the CDK4/6 inhibitor, which is deleted or mutated in 40% of melanomas, and the PI3K/AKT pathway inhibitor, which exhibits characteristic loss of function in 20–30% of melanomas, were considered. TP53, a regulator of the cell cycle and apoptosis, frequently mutated in advanced human melanomas, and NF1, a RAS inhibitor with mutations present in 10–15% of human melanomas, were also included. As a result of this analysis, MITF was found to be upregulated with a log2FC of 2.86 and an extremely significant *p*-value. NF1 was also upregulated with a log2FC of 1.17 and a significant *p*-value (0.001).

### 3.8. Go and KEGG Analysis

**Figure 5:**
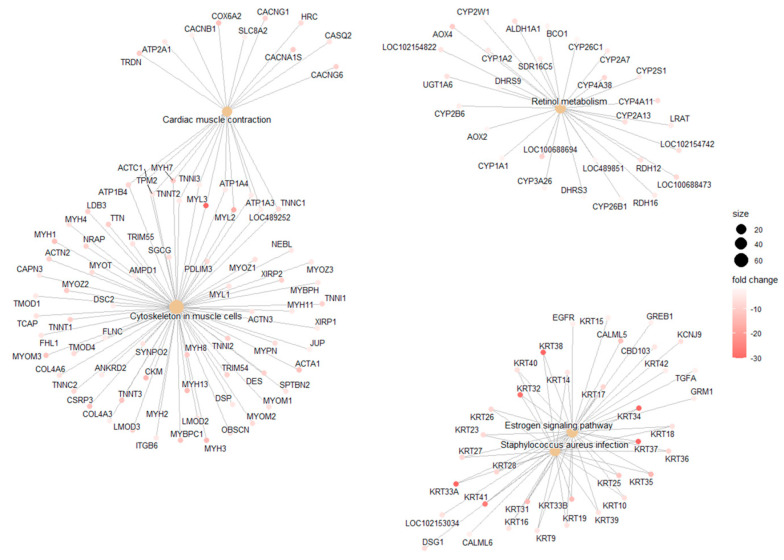
Pathway Enrichment Analysis of DEGs Identified by DESeq2.

GO analysis categorizes DEGs obtained in the DESq2 analysis into three categories as follows: (i) Biological Process (BP); (ii) Cellular Component (CC); and (iii) Molecular Function (MF). GO identified 10 BP, 8 MF, and 8 CC DEGs, and dotplots of the results are shown in Figure 4B–D. Pathway analysis showed six common pathways that were significantly enriched (Figure 5).

Pathway analysis of DEGs derived from the DESeq2 workflow using curated databases. Enriched biological pathways provide insight into the molecular mechanisms underlying melanoma pathology.

### 3.9. Isoform Analysis and DEGs Overlaps

**Figure 6:**
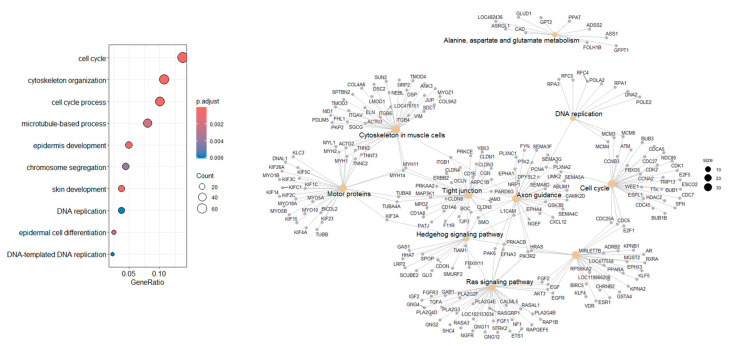
Functional and pathway analysis of HISAT2–StringTie–Ballgown-derived DEGs. Combined Gene Ontology and pathway enrichment analysis of DEGs identified by the HISAT2–StringTie–Ballgown pipeline.

Ballgown transcript-level analysis identified 2366 transcripts without known gene annotations, while 1716 were successfully matched. Common DEGs between Ballgown and DESeq2 were analyzed for functional enrichment using GO and KEGG (Figure 6). This highlights the potential identification of novel transcript isoforms relevant to COM biology.

### 3.10. RT-PCR Determinations

**Figure 7:**
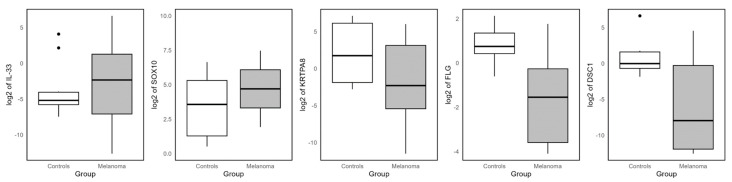
RT-qPCR validation of common DEGs identified by both workflows. Boxplots showing the RT-qPCR expression levels of two commonly upregulated and three commonly downregulated genes across independent melanoma and control samples. These genes were initially identified as differentially expressed by both the Bowtie2–DESeq2 and HISAT2–StringTie–Ballgown RNA-seq workflows.

To validate the RNA-seq results, RT-qPCR was performed on a subset of DEGs in an independent set of melanoma and control samples. Five genes were selected for validation as follows: two upregulated (IL-33 and SOX10) and three downregulated genes (KRTPA8, FLG and DSC1) identified by both RNA-seq workflows (Figure 7). The expression patterns obtained by RT-qPCR were consistent with the RNA-seq data, confirming the differential expression in melanoma samples relative to controls. Notably, GATA4, although identified as the most upregulated gene in the Bowtie2–DESeq2 analysis, was excluded from the validation due to inefficient amplification.

## 4. Discussion

### 4.1. Uncharacterized Genes and Contextual Complexity

Our results show that a significant percentage of differentially expressed genes (DEGs) obtained in DESeq2 analysis (43% of upregulated and 51% of downregulated genes) remain uncharacterized. Our results show that 43% of upregulated and 51% of downregulated DEGs obtained in the DESeq2 analysis remain uncharacterized, highlighting a substantial knowledge gap in the functional annotation of canine genes. This lack of genetic information reflects the limited understanding of their function and suggests that many relevant biological pathways in the canine oral melanoma (COM) model may still be unexplored. Furthermore, gene expression responses, whether upregulated or downregulated, can be influenced by various factors, such as cellular context, including tissue type or the tumor microenvironment [13,14]; environmental conditions, such as oxidative stress or inflammation [15,16,17]; or and epigenetics regulation, including DNA methylation and histone modifications [16,17,18,19], making it difficult to characterize some genes adequately in the appropriate context [20].

### 4.2. Upregulated Genes

In our Bowtie2-DESeq2 analysis, GATA4 was identified as the most upregulated gene in COM samples compared to healthy tissue. GATA4 is a transcription factor crucial for cell differentiation and survival in various tissues [21]. However, overexpression could not be validated by RT-PCR due to inefficient amplification. This discrepancy may stem from technical issues such as suboptimal primer design or RNA degradation, or it could be reflecting biological variability. Further studies are needed to clarify the expression and potential role of GATA4 in canine oral melanoma. The following three upregulated genes LOC111096139, LOC100686948, and LOC11091077 are presumably genes that lack clear annotation in common databases. This suggests that it could be orthologs from another species or transcripts with an uncharacterized function. The fourth gene was SALL3, a gene belonging to the SALL gene family, encoding transcription factors involved in embryonic development. In humans, SALL3 is not considered a significant prognostic marker [22], and its overexpression in COM could suggest a role in tumor progression, although further research is needed to confirm this hypothesis.

### 4.3. Downregulated Genes

The four most significantly downregulated genes in COM, compared to healthy tissue controls, were all keratin genes (KRT33A, KRT86, KRT34, and KRT38), which is consistent with a potential loss of epithelial differentiation, a hallmark of aggressive tumor behavior [20,23,24]. Keratins are structural proteins essential for epithelial integrity and cellular differentiation. Their downregulation is associated with a shift toward a mesenchymal-like phenotype, facilitating tumor cell migration and invasion—hallmarks of the epithelial–mesenchymal transition (EMT), a key mechanism by which epithelial cells acquire mesenchymal characteristics and increase their motility and invasiveness [25]. In human cutaneous melanoma, keratin downregulation has been linked to increased malignancy and reduced cell adhesion [26]. The drastic fold change observed in our study (log2FoldChange of −30) highlights a substantial shift away from epithelial characteristics, potentially indicating a more aggressive tumor phenotype.

### 4.4. Ballgown Analysis Results

Using the Ballgown pipeline, the most highly upregulated gene was IL33, followed by SOX10, SCG3, and SHC4. Among the first downregulated genes were KRT1, KRTDAP, FLG, and LOC607095.

IL-33, an alarmin of the IL-1 family, is involved in inflammation and immune cell activation [27]. Although the impact of IL-33 in immune cell function in melanoma remains unclear, it has been shown to foster a tumor microenvironment that promotes melanoma growth [28,29,30]. SOX10 is a key transcription factor in melanocyte biology and melanoma development, and it tends to be homogeneously expressed in most melanomas [31,32,33]. SCG3 is related to the secretion of vesicles and exosomes [34]. It has been implicated in the progression of certain human cancers [35,36] and may contribute to the spread of canine melanoma. SHC4 acts in cell signaling pathways, including the activation of MAPK/ERK, which is key in melanoma proliferation [37,38]. Its overexpression suggests a role in tumor growth and possibly in therapy resistance [38].

Among the genes downregulated in COM in Balgwon analysis, the KRT1 gene was identified as the most significantly downregulated. This implicates this keratin in the loss of normal epithelial characteristics [26]. In addition, KRTDAP, a gene associated with keratinocyte differentiation, is repressed. Its repression is indicative of dedifferentiation, common in tumors that are more aggressive [39]. FLG encodes a key structural protein in the epithelial barrier [40]. Its decrease could be related to increased cell plasticity and melanoma progression [40,41]. LOC607095 is a possible gene and its human ortholog could be of interest in melanoma; however, its function is unknown or uncharacterized in canines.

### 4.5. Comparison Between Methods

The genes identified by both the DESeq2 and Ballgown approaches showed a high degree of consistency, with their relative positions aligning closely. The similarity of DEGs between the approaches was especially strong among the downregulated genes. For instance, all ten downregulated genes found by DESeq2 were also identified by Balgown, with ZSCAN2, IL33, and SOX10 ranking highly in both lists. On the other hand, nine of the top ten downregulated genes in Balgown were shared with DESeq2. This consistency strengthens the validity of our results and suggests these genes are strong candidates for further functional studies and potential biomarkers.

ZSCAN2 is a zinc finger family transcription factor with a possible role in gene regulation [42]. Its function in melanoma is not well characterized. Still, it could be involved in the regulation of genes associated with cell differentiation and with the control of tumor proliferation and survival. SOX10 activates the MITF (microphthalmia-associated transcription factor) pathway, which controls genes crucial for melanoma survival and its overexpression is associated with increased tumor aggressiveness and resistance to therapies. The overexpression of the IL33 gene in both methods further supports its potential role in melanoma progression. ESM1 encodes for endothelial cell-specific molecule 1, also known as endocan; is the next most common gene, and it is regulated by cytokines [43].

### 4.6. Comparison with Previous Studies

Several previous transcriptomic studies have identified common and divergent patterns in COM. Fowles et al. [44] found that COM exhibits molecular pathway alterations similar to those observed in human melanoma, including activation of the MAPK pathway which aligns with our detection of BRAF and NRAS. Our findings on MITF and NF1 further suggest that certain COM subtypes share mechanisms with human melanomas [45]. Additionally, the molecular heterogeneity of COM has been documented in previous studies, identifying subtypes with profiles similar to human melanomas with BRAF mutations or NF1 deficiency [13]. Our analysis showed modest NF1 overexpression, suggesting that the cases analyzed may not follow the same NF1 loss pattern observed in human melanomas.

Pisamai et al. [46] performed transcriptome sequencing of early-stage and late-stage COM and observed both similarities and discrepancies with our findings, likely because of differences in sample selection or methodologies. Rahman et al. [20], whose transcriptomic analysis of COM revealed both similarities and differences with human melanoma, emphasize the need for more standardized approaches to understand the complex molecular landscape of COM. Ploypetch et al. [47] studied salivary proteomics in COM and found that the ratio of free ubiquitin D (fUBD) to conjugated ubiquitin D (cUBD) could serve as a prognostic biomarker for survival.

### 4.7. Chromosomal Distribution and Oncogenes

Our analysis revealed an asymmetrical distribution of DEGs across chromosomes. This pattern may reflect the genomic organization of coding genes in the canine genome, where larger chromosomes generally contain more genes. Furthermore, the significant number of DEGs observed on chromosome 9 suggests its potential involvement in the biological response to COM. Similar chromosomal patterns have been reported in previous studies [13,20].

In the search for human-relevant oncogenes and tumor suppressor genes, MITF was found to be upregulated in the dataset, a result considered to hold significant relevance and potentially offering valuable insights into the COM model. MITF is a key oncogene in human melanoma, where it plays a critical role in melanogenesis, melanocyte survival, and tumor progression [48].

### 4.8. Gene Ontology and Pathway Analysis

Gene Ontology (GO) analysis revealed genes involved in biological processes such as cell differentiation and transcription regulation. KEGG pathway analysis highlighted several metabolic and signaling pathways affected, including cardiac muscle contraction, retinol metabolism, estrogen signaling, and Staphylococcus aureus infection. These findings align with previously published research on COM which identified angiogenesis and Wnt/β-catenin signaling as significantly enriched pathways [49]. This analysis also aligns with metabolomic studies in dogs with oral melanoma, which identified alterations in amino acid metabolism that may be linked to metabolic shifts necessary for tumor growth [47,50].

### 4.9. Study Limitations

While our transcriptomic analysis into COM provides valuable insights, several limitations should be acknowledged. First, the relatively small sample size (*n* = 6 per group) may not capture the full molecular heterogeneity of COM and reduces the statistical power for subgroup analyses, emphasizing the need for larger and more diverse cohorts. Second, the absence of functional validation restricts the interpretation of DEGs and future in vitro and in vivo studies are needed. Third, reliance on reference-based transcriptome assembly may introduce bias, and alternative approaches like long-read sequencing could reveal additional insights. Fourth, the large proportion of uncharacterized genes among the differentially expressed transcripts limits the functional interpretation and translational potential of the results. While this issue partly stems from the intrinsic biological complexity of melanoma, it also reflects the current limitations of the canine genomic resources. In particular, the use of the most recent reference genome, CamFam6, though beneficial in terms of improved assembly and coverage, is still affected by a significant number of genes lacking formal annotation or clear orthology with better-studied species. Additionally, nested or incomplete gene models further complicate gene identification and downstream pathway analysis. These challenges underscore the need for continued improvement in canine genome annotation efforts and integration of complementary functional studies, for example, proteomics or in vitro validation, to better characterize these genes in future works. Fifth, focusing on the transcriptomic changes and the integration of proteomics or metabolomics would provide a more comprehensive view of COM biology. Lastly, pathway analysis is constrained by existing database annotations and may not fully capture tumor-specific mechanisms.

Given these constraints, this work should be considered a pilot study aimed at generating hypotheses and guiding future research. Ongoing efforts in our laboratory include expanding the sample cohort, performing protein validation, and conducting functional assays in vitro to characterize the oncogenic potential of key candidate genes.

## 5. Conclusions

Using Bowtie2-DESeq2 analysis, a significant proportion of differentially expressed genes (DEGs) in COM were identified as uncharacterized, highlighting gaps in genetic knowledge and potential unexplored pathways. Given the complex regulation of gene expression, several key upregulated genes were found, including GATA4, SALL3, IL33, SOX10, and SCG3/SHC4, while the downregulation of keratin genes suggests a loss of epithelial differentiation, contributing to aggressive tumor behavior. This observation may reflect the activation of epithelial–mesenchymal transition (EMT) programs, a process known to enhance tumor cell motility, invasiveness, and metastatic potential.

The strong concordance between Bowtie2-DESeq2 and HISAT-StringTie-Ballgown analyses reinforces the reliability of the findings, with ZSCAN2, IL33, and SOX10 emerging as potential biomarkers. Molecular parallels between COM and human melanoma, particularly MAPK pathway alterations and genes like BRAF, NRAS, MITF, and NF1, further support this study’s relevance. Notably, MITF appears to play a crucial role in tumor progression and therapy resistance.

The asymmetric chromosomal distribution of DEGs, particularly on chromosome 9, suggests underlying genomic influences on COM. GO and KEGG analyses reveal disruptions in pathways related to cell differentiation, transcriptional regulation, and immune response, while metabolomic data indicate shifts in amino acid metabolism associated with melanoma progression.

These findings highlight IL33, SOX10, ZSCAN2, and MITF as promising candidates for further research and potential therapeutic exploration.

## Figures and Tables

**Table 1:** Main characteristics of studied patients.

ID	Sex	Age	Breed	Tumor Location	Size	TNM
Control_1	Male	6	American Stafford	-	-	-
Control_2	Female	5	Mix Stafford	-	-	-
Control_3	Female	9	Pitbull	-	-	-
Control_4	Female	8	Pitbull	-	-	-
Control_5	Male	11	Mix Pitbull	-	-	-
Control_6	Female	5	Mix Stafford	-	-	-
Melanoma_1	Female	10	American Staffordshire Terrier	Lip	2 cm	Stage II
Melanoma_2	Female	10	Chihuahua	Mandible	3.5 cm	Stage III
Melanoma_3	Male	8	Canary Mastiff	Cheek	7.7 cm	Stage III
Melanoma_4	Male	14	Yorkshire Terrier	Lip	2.1 cm	Stage II
Melanoma_5	Female	12	Mixed	Lip	1.5 cm	Stage I
Melanoma_6	Male	12	Schnauzer	Maxilla	3.8 cm	Stage III

TNM stands for tumor, node, and metastasis.

**Table 2:** Chromosome location and mean expression of the top identified differentially expressed genes in canine oral melanoma and healthy control tissue samples.

Gene Name	Chromosome	Start Position	End Position	Mean Control	Mean Melanoma
ABCC11	2	67,365,969	67,434,568	1,206,093,988	513,458,128
ATRNL1	28	26,352,303	27,138,636	8,453,868,178	107,012,498
CASZ1	2	85,844,387	85,966,963	1,145,693,802	5,715,992,023
CEP170	7	34,091,156	34,222,655	1,065,593,156	1,303,246,121
CES1	2	60,780,757	60,850,933	1,199,658,343	4,492,333,331
COL17A1	28	16,828,097	16,880,431	144,398,744	867,508,665
DSC1	7	58,308,188	58,387,461	1,286,131,338	4,125,987,355
ETV1	14	28,575,516	28,670,323	8,799,008,458	111,593,121
FAT2	4	58,185,984	58,263,273	126,269,534	8,691,156,552
FOLH1B	21	10,581,865	10,647,452	8,524,754,322	1,171,190,646
GATA4	25	26,280,510	26,337,654	3,825,626,408	4,758,170,722
IL22RA1	2	76,014,607	76,034,628	951,117,109	4,381,898,873
IL33	11	28,069,663	28,108,194	967,667,444	1,412,622,417
KRT34	9	22,098,153	22,101,234	9,432,768,672	3,825,626,408
KRT86	27	2,656,611	2,826,996	1,003,341,814	3,825,626,408
KRTAP11-1	31	25,995,174	25,995,635	838,372,259	3,825,626,408
MMP16	29	34,120,818	34,403,822	8,285,952,144	1,029,971,631
MYEF2	30	14,532,118	14,564,995	9,328,405,867	1,145,666,304
PCSK1N	X	42,166,878	42,172,740	4,946,786,732	7,722,763,719
POU2F3	5	13,632,917	13,707,369	9,638,707,384	4,926,011,945
PRKACB	6	64,155,572	64,269,867	1,075,963,493	1,234,943,757
SBSN	1	117,733,283	117,738,080	1,294,205,254	6,188,281,965
SDSL	26	10,990,071	11,015,079	4,681,131,408	7,338,404,409
SHC4	30	15,185,863	15,322,777	8,292,860,068	1,262,090,089
SKA3	25	17,118,211	17,138,178	7,694,448,604	9,481,288,937
SLF1	3	14,670,381	14,744,679	9,059,277,209	1,07,288,861
TENM3	16	49,667,658	50,270,179	929,343,987	1,298,114,629
TP63	34	21,700,663	21,918,660	1,302,620,425	7,773,881,578

**Table 3:** Top 10 upregulated and top 10 downregulated differentially expressed genes in canine oral melanoma and healthy skin tissue samples resulting from the DESeq2 analysis.

Gene	Description	Log2 Fold Change	*p*-Value Adjusted
GATA4	GATA binding protein 4	2,178,905,058	4.79 × 10^−11^
LOC111096139	Uncharacterized	9,282,033,508	3.05 × 10^−7^
LOC100686948	Serine/arginine-rich splicing factor SR45-like	9,089,771,615	3.11 × 10^−11^
LOC111091077	Uncharacterized	8,810,707,393	0.016373206
SALL3	Spalt-like transcription factor 3	844,175,455	0.017528697
LOC119867902	Uncharacterized	8,421,755,882	2.86 × 10^−6^
LOC119869412	Uncharacterized	8,300,275,074	4.87 × 10^−8^
LOC612108	Uncharacterized	7,933,603,842	0.000639468
LOC111090349	Uncharacterized	7,525,602,976	9.04 × 10^−7^
CDH18	Cadherin 18	7,448,216,395	5.22 × 10^−5^
KRT33A *	Keratin 33A	−30	1.07 × 10^−27^
KRT86 *	Keratin 86	−30	2.10 × 10^−27^
KRT34 *	Keratin 34	−30	3.76 × 10^−27^
KRT37 *	Keratin 37	−30	1.77 × 10^−20^
KRT38	Keratin 38	−30	1.77 × 10^−20^
LOC102151404 *	Keratin, high-sulfur matrix protein, B2A-like	−2,933,651,986	1.43 × 10^−19^
KRTAP11-1 *	Keratin-associated protein 11-1	−285,367,828	1.30 × 10^−26^
KRTAP8-1 *	Keratin-associated protein 8-1	−2,853,215,131	1.70 × 10^−18^
KRT32 *	Keratin 32	−2,809,598,496	2.72 × 10^−18^
LOC102151268 *	Keratin-associated protein 1-3-like	−2,801,190,653	4.40 × 10^−24^

* Genes in common with Ballgown analysis.

**Table 4:** Top 10 upregulated and top 10 downregulated differentially expressed genes in canine oral melanoma and healthy skin tissue samples (Ballgown analysis).

Gene	Description	Log Fold Change	q-Value ^#^
IL33 *	Interleukin 33	4.0 × 10^14^	0.008
SOX10	SRY-box transcription factor 10	4.0 × 10^14^	0.041
SCG3	Secretogranin III	4.0 × 10^14^	0.044
SHC4	SHC adaptor protein 4	3.0 × 10^14^	0.015
CDH19	Cadherin 19	3.0 × 10^14^	0.027
TENM3	Teneurin transmembrane protein 3	3.0 × 10^14^	0.017
FOLH1B	Folate hydrolase 1B	3.0 × 10^14^	0.024
ESM1 *	Endothelial cell-specific molecule 1	3.0 × 10^14^	0.048
LOC119867250	U6 spliceosomal RNA	3.0 × 10^14^	0.019
ARHGAP24	Rho GTPase activating protein 24	3.0 × 10^14^	0.046
KRT1 *	Keratin 1	−9.680895	0.010
KRTDAP *	Keratinocyte differentiation associated protein	−8.004239	0.016
FLG *	Filaggrin	−7.937734	0.016
LOC607095 *	Lysine-rich arabinogalactan protein 19-like	−7.715431	0.013
KRT17 *	Keratin 17	−7.653794	0.0001
CALML5 *	Calmodulin-like 5	−7.545614	0.041
KRT10 *	Keratin 10	−6.935247	0.024
PIP *	Prolactin-induced protein	−6.912647	0.004
DSC1 *	Desmocollin 1	−6.702341	0.037
TNNI2 *	Troponin I2, fast skeletal type	−6.613600	0.037

* Genes in common with DESeq2 analysis. ^#^ In Ballgown, the q-value represents the adjusted *p*-value, calculated using the Benjamini–Hochberg method or similar approaches to control the false discovery rate (FDR).

## Data Availability

The RNA-seq data generated in this study are part of an ongoing collaboration between two institutions. The data will be made publicly available upon agreement and publication. Until then, they are available from the corresponding authors upon reasonable request.
